# Allergenarrays: low-cost food allergy diagnosis using allergen extracts-based microarrays

**DOI:** 10.1186/2045-7022-4-S2-P37

**Published:** 2014-03-17

**Authors:** Jean-Philippe Borges, Camille Gironde, Lidwine Trouilh, Benjamin Berteloite, Andre Pol Apoil, Pierre Rouge, Annick Barre, Emmanuelle Trevisiol

**Affiliations:** 1 INSA de Toulouse, LISBP, Toulouse, France; 2 Faculte de Pharmacie, Toulouse III, PharmaDev, Toulouse, France; 3 Innopsys, R&D, Carbonne, France; 4 Hopital de Rangueil Laboratoire d'Immunologie, Toulouse, France

## Background

The current tools available for diagnosing food allergy include the clinical history, trial elimination diets, diet diaries, skin prick testing (SPT) and allergen-specific serum IgE testing. Since 15 years, development of allergen-based microarrays using recombinant/natural proteins is increasing, resulting in commercially available microarrays to detect specific IgE against more than 100 allergenic compounds. For the moment, the number of available recombinant allergens and the cost of their development limit the enhancement of allergen microarray technology.

The goal of our study is to explore and optimize allergen-microarray from food allergen extracts and trying to create a low-cost IgE detecting platform.

## Method

One of the most critical step is the preparation of food extracts containing all of the major allergens. To standardize and miniaturize the sample preparation, high-speed benchtop homogenizer offers the ultimate performance for the lysis of biological samples. In some cases, anti-protease cocktails were added to increase allergen concentrations. Food extracts are deposited using a microarrayer device on glass slides at different concentrations, offering a range for further IgE interactions. IgE-binding was detected using fluorescent anti-human IgE antibody. Using fluorescent antibodies with different fluorochromes in the detecting step improve the versatility of the experiment, allowing to obtain a huge amount of data from a single experiment.

## Results

Our food extracts-based microarrays for food allergen diagnosis are currently investigated for a large number of patients. The first results showed a good correlation with previous existing conventional assays currently used in allergy diagnosis. The best results were obtained for the diagnosis of peanut, milk, egg and nuts (walnut, hazelnut, Brazil nut, pecan, almond...). Fish, cereals and fruits allergy extracts need to be improved regarding stability and allergen concentration. Further developments are needed to enhance allergen stability and coating on slides.

## Conclusion

Food extracts-based microarrays are efficient tools for allergy diagnosis and need to be improved, using different type of allergens. Using food extracts instead of recombinant proteins could be a good alternative to design low-cost microarrays.

